# Reduced frontal-subcortical white matter connectivity in association with suicidal ideation in major depressive disorder

**DOI:** 10.1038/tp.2016.110

**Published:** 2016-06-07

**Authors:** W Myung, C E Han, M Fava, D Mischoulon, G I Papakostas, J-Y Heo, K W Kim, S T Kim, D J H Kim, D K Kim, S W Seo, J-K Seong, H J Jeon

**Affiliations:** 1 Department of Psychiatry, Depression Center, Samsung Medical Center, Sungkyunkwan University School of Medicine, Seoul, Korea; 2 Department of Biomedical Engineering, Institute of Health Science, Korea University, Seoul, Korea; 3 Department of Electronics and Information Engineering, Korea University, Sejong, Korea; 4 Depression Clinical and Research Program, Massachusetts General Hospital, Harvard Medical School, Boston, MA, USA; 5 Department of Radiology, Samsung Medical Center, Sungkyunkwan University School of Medicine, Seoul, Korea; 6 Department of Neurology, Samsung Medical Center, Sungkyunkwan University School of Medicine, Seoul, Korea; 7 Department of Health Sciences and Technology, Department of Medical Device Management and Research, Department of Clinical Research Design and Evaluation, Samsung Advanced Institute for Health Sciences and Technology, Sungkyunkwan University, Seoul, Korea

## Abstract

Major depressive disorder (MDD) and suicidal behavior have been associated with structural and functional changes in the brain. However, little is known regarding alterations of brain networks in MDD patients with suicidal ideation. We investigated whether or not MDD patients with suicidal ideation have different topological organizations of white matter networks compared with MDD patients without suicidal ideation. Participants consisted of 24 patients with MDD and suicidal ideation, 25 age- and gender-matched MDD patients without suicidal ideation and 31 healthy subjects. A network-based statistics (NBS) and a graph theoretical analysis were performed to assess differences in the inter-regional connectivity. Diffusion tensor imaging (DTI) was performed to assess topological changes according to suicidal ideation in MDD patients. The Scale for Suicide Ideation (SSI) and the Korean version of the Barrett Impulsiveness Scale (BIS) were used to assess the severity of suicidal ideation and impulsivity, respectively. Reduced structural connectivity in a characterized subnetwork was found in patients with MDD and suicidal ideation by utilizing NBS analysis. The subnetwork included the regions of the frontosubcortical circuits and the regions involved in executive function in the left hemisphere (rostral middle frontal, pallidum, superior parietal, frontal pole, caudate, putamen and thalamus). The graph theoretical analysis demonstrated that network measures of the left rostral middle frontal had a significant positive correlation with severity of SSI (*r*=0.59, *P*=0.02) and BIS (*r*=0.59, *P*=0.01). The total edge strength that was significantly associated with suicidal ideation did not differ between MDD patients without suicidal ideation and healthy subjects. Our findings suggest that the reduced frontosubcortical circuit of structural connectivity, which includes regions associated with executive function and impulsivity, appears to have a role in the emergence of suicidal ideation in MDD patients.

## Introduction

Suicide is one of the major causes of death. Over 800 000 people die from suicide every year, and many more attempt suicide worldwide.^1^ Patients with major depressive disorder (MDD) represent a population with high suicide risk, with 16% reporting at least one suicide attempt during their lifetime. The risk of completed suicide in patients with MDD is estimated at 3.4–6.8% (ref. 2), ~30 times greater than that of the general population.^3^

Many studies have investigated factors associated with completed suicide or attempts in MDD patients, such as psychiatric comorbidities,^4^ biomarkers,^5, 6^ environmental factors^7^ and traumatic experience.^8, 9^ In addition, impulsivity has been reported to be associated with suicidal behavior across numerous studies.^10^ MDD patients with suicidal ideation have a higher rate of previous suicide attempts,^11^ have a lower treatment response rate and take longer to respond to treatment.^12^ Although previous studies have suggested that MDD patients with suicidal ideation differ biologically from those without suicidal ideation,^13, 14^ few studies have specifically focused on the differences in brain networks between MDD patients with and without suicidal ideation.

Recent neuroimaging studies also suggest that structural or functional changes in the brain may be involved in suicide attempts in MDD. Several structural magnetic resonance imaging (MRI) studies^15^ demonstrated that periventricular white matter hyperintensities increased in MDD patients with histories of suicide attempts. Moreover, a number of neuroimaging studies utilizing structural MRI^16^, functional MRI^17^, single-photon emission computed tomography,^18, 19^ position emission tomography^20^ and diffusion tensor imaging (DTI)^21, 22^ have reported convergent findings involving the structure or function of frontal neural systems in suicide attempters.^23, 24^ However, none of these studies investigated alterations of brain networks in MDD patients with suicidal ideation. Suicidal ideation is the first step on the path to suicide, and it is also a phenomenon distinct from suicide attempts or suicidal behaviors.^25^ In addition, suicidal ideation does not simply represent depression severity, but rather it is a distinct phenomenon that differs from other depressive symptoms in underlying biology, impacts on impairment, and risk factors.^26^ Alterations in the brain networks of MDD patients with suicidal ideation are important for understanding the development of risk for suicide attempts in MDD.

Graph theoretical analysis models the whole brain as a network, which consists of nodes corresponding to brain regions, and edges representing the relationship between any pair of brain regions, and has been used to investigate the organizational changes of brains in patients.^27, 28, 29^ One of the primary research interests of graph theoretical analysis is localizing abnormal brain connectivity. Network-based statistics (NBS) analysis^30^ offers a powerful and complementary approach for this purpose and was found to be successful in clinical applications.^31^ The graph theoretical measures of white matter brain networks also provide valuable information about disturbances in the network organization. This advanced approach has been used successfully in studies of mental disorders.^32, 33^

In this study, we performed a NBS analysis and graph theoretical analysis using DTI to investigate the topological organization of whole-brain white matter networks according to suicidal ideation in MDD patients. We sought to determine (1) whether MDD patients with suicidal ideation show different topological efficiency and nodal/connectional properties in the white matter networks compared with MDD patients without suicidal ideation, (2) whether these topological changes significantly correlate with suicidal ideation or impulsivity and (3) whether the structural connectivity associated with suicidal ideation in MDD patients differs between MDD patients and healthy subjects.

## Materials and methods

### Subjects

We enrolled the patients between April 2011 and April 2013. A total of 49 MDD patients (five male and 44 female patients) were recruited from the outpatient clinic of the Depression Center of the Samsung Medical Center. The 49 patients comprise 24 MDD patients with suicidal ideation and 25 age-, gender- and education year-matched MDD patients without suicidal ideation. No patients received psychotropic medication at the baseline visit. In addition, no patients received psychotropic medication within 2 weeks of the study or fluoxetine within 4 weeks. Inclusion criteria were age ⩾18 years, current unipolar major depressive episode, as verified by Diagnostic and Statistical Manual of Mental Disorders Fourth Edition criteria for MDD,^34^ and scores ⩾16 on the 17-item the Hamilton Depression Rating Scale (HAM-D).^35^ The diagnosis was based on clinical evaluation by a board-certified psychiatrist and the full version of the Mini-International Neuropsychiatric Interview,^36^ which was applied by one psychologist blinded to the study. Exclusion criteria were any psychotic disorder (for example, schizophrenia or delusional disorder), bipolar affective disorder, neurological illness including significant cognitive impairment or Parkinson's disease, mental retardation, significant medical conditions, epilepsy, history of dependence on alcohol or drugs, personality disorders or brain damage. No patients had history of non-suicidal self-harm behaviors in their current depressive episodes.

In addition, 31 healthy volunteers with no history of psychiatric disease were recruited from advertisements. Volunteers with a positive family history of mood disorder were excluded. The study protocol was approved by the ethics review board of Samsung Medical Center, Seoul, Korea. Signed informed consent was obtained from all participants.

### Clinical evaluation

At entry, suicidal ideation was assessed with the suicidality module of the Mini-International Neuropsychiatric Interview^36^ Patients answering ‘yes' to the question ‘In the past month did you think about suicide?' were classified as the ‘suicidal ideation group' (*n*=24), and those answering ‘no' were classified as the ‘no suicidal ideation group' (*n*=25).

The severity of suicidal ideation was assessed with the Scale for Suicide Ideation (SSI),^37^ a 19-item scale designed to measure the intensity, pervasiveness and characteristics of suicidal intent. The Korean version of the Barrett Impulsiveness Scale (BIS) was used to assess self-report impulsivity.^38^ The Korean version of BIS has 23 items divided into three subscales: motor impulsivity (for example, ‘I am restless at lectures or conversation'), attention-cognitive impulsivity (for example, ‘I get easily bored when solving thought problems') and non-planning impulsivity (for example, ‘I spend or charge more than I earn'). The severity of depression was measured using the 17-item HAM-D.^35^ The Mood Disorder Questionnaire (MDQ)^39^ was used to detect bipolarity in depressed patients.^40^ HAM-D and MDQ were administered by a single trained rater.

### Image acquisition

T1 and diffusion-weighted images were acquired from all 49 patients and 31 healthy subjects using the same 3.0-T MRI scanner (Philips 3.0 T Achieva) within 1 week after the baseline visit. T1-weighted MRI data were recorded using the following imaging parameters: 1 mm sagittal slice thickness, overcontiguous slices with 50% overlap, no gap, repetition time (TR) of 9.9 ms, echo time (TE) of 4.6 ms, flip angle of 8° and matrix size of 240 × 240 pixels. Images were reconstructed to 480 × 480 over a 240-mm field of view. In the whole-brain diffusion-weighted MRI examination, sets of axial diffusion-weighted single-shot echo-planar images were collected with the following parameters: 128 × 128 acquisition matrix, 1.72 × 1.72 × 2 mm^3^ voxels reconstructed to 1.72 × 1.72 × 2 mm^3^, 70 axial slices, 220 × 220 mm^2^ field of view, TE 60 ms, TR 7383 ms, flip angle 90°, slice gap 0 mm and b-factor of 600 s mm^−2^.With the baseline image without diffusion weighting (the reference volume), diffusion-weighted images were acquired from 45 different directions. All axial sections were acquired parallel to the anterior commissure-posterior commissure line.

### Image preprocessing and network construction

Freesurfer was used to obtain surface meshes of the boundary between the gray and white matter from T1 anatomical brain images (http://surfer.nmr.mgh.harvard.edu). After registering surface meshes into the DTI space, volumetric regions of interests (ROIs) were generated based on the Desikan atlas, which includes gray matter voxels of 34 anatomical regions of cortices^41, 42^ and seven subcortical regions^41^ for each hemisphere. Thus, we obtained 82 ROIs as nodes of the brain networks.

To obtain streamline tractography from eddy-current-corrected diffusion-weighted images (FSL, http://www.fmrib.ox.ac.uk/fsl/), we used the Fiber Assignment by Continuous Tracking (FACT) algorithm,^43^ with 45 degrees of angle threshold and eight random seed per voxel through the Diffusion toolkit along with TrackVis.^44^ This program performed tractography from the all voxels (seed voxels) of white matter, except ventricles. We obtained connectivity matrices from the defined and registered ROIs and tractography, counting the number of streamlines between all pairs of defined ROIs using the UCLA Multimodal Connectivity Package (http://ccn.ucla.edu/wiki/index.php). The resulting matrix contains the streamline count between all pairs of ROIs as its weight.

### Network topological measures

Network topological measures of nodes were computed using Matlab routines of the Brain Connectivity Toolbox.^45^ The measures include nodal degree, nodal strength, clustering coefficient, participation coefficients, regional efficiency and betweenness centrality. The nodal degree and strength captured how many neighbors a node is connected with and how strongly it is connected with its neighbors, respectively. The former is the number of edges that are connected to the node, whereas the latter is the summation of the edges' strengths in terms of the number of streamlines. The clustering coefficient of a node represents how densely its neighbors are connected: the higher value indicates that its neighbors are clustered together centered with the node.^46^ The participation coefficient of a node captures its role in the modular organization.^47^ The higher value represents that the node is connected with multiple modules and may have an important role in exchanging information between modules. ^46^ The regional efficiency summarizes how efficiently the information of a node can be exchanged with all the other nodes by averaging reciprocals of the shortest path lengths to all the other nodes.^48^ The betweenness centrality of a node captures its importance in the network by counting the number of shortest paths that pass through the node.^45, 49^ The node with higher centrality is more important in the network in terms of overall information exchange.

### Statistical analysis

Categorical variables are summarized as frequencies and proportions. Continuous variables are presented as mean±s.d. or as the median and interquartile range, and Student's *t*-test, one-way analysis of variance, Wilcoxon rank-sum test or Kruskal–Wallis test was used according to the normality of the distribution.

We first performed the NBS analysis^30^ between structural brain networks of MDD patients with and without suicidal ideation in order to detect a subnetwork that was significantly different between the two groups. NBS extended the widely used cluster analysis in voxel-based morphometry^50^ to networks and insisted that a subnetwork, a set of connected abnormal edges, was more responsible for abnormal behaviors than a single abnormal edge is. Thus, as the cluster analysis does, it acted as a more statistically powerful multiple comparison correction procedure for massive univariate tests; in our case, for ~3300 edges.

In short, we first compared networks of MDD patients with suicidal ideation and without the ideation using two-sample *t*-test edge-by-edge, and then employed NBS for multiple comparison correction. NBS estimated significance levels of subnetworks based on how the size of the subnetworks (clusters) was bigger than randomly formed subnetworks using permutation testing, where a cluster was defined as a set of connected edges whose edge weight was bigger than a certain initial threshold; we used 2.2 as the initial threshold. Specifically, we re-populated the data sets *N*−1 times by random re-assignment (permutation) of all subjects into one of two groups, where *N* is the number of permutations. We computed maximum size of subnetworks for the original data set and *N*−1 permuted sets, which formed a null distribution of sizes of the randomly formed subnetworks. Then, we estimated the significance level by a fraction of the occurrence whose sizes of the randomly formed subnetworks were not less than the size of the subnetwork of the original data set. We used 10 000 as *N*.

We extracted hub regions of the identified subnetworks, whose degree exceeded the mean and two s.d.'s over all regions connected by the edges found by NBS. The hub regions, in general, represent the brain regions with great influence to other regions in the network because of their dense connections. We performed the correlation test between clinical measurement and the various nodal measures of the hub regions. As the network measures often do not follow the normal distribution, we used the Spearman partial correlation to control for effects of age, gender and level of education. We then performed the false discovery rate (FDR) procedure^51^ for the multiple comparison correction over hub nodes.

Next, we investigated the total connectivity (cumulative number of streamlines) in the identified subnetwork. The total connectivity is computed as the total number of streamlines over all edges in the identified subnetwork. In this experiment, we compared the total connectivity not only between patient groups but also between MDD patients and healthy subjects. We employed permutation-based analysis of covariance for three groups, controlling for the effects of age, gender and level of education. Similar to the permutation procedure in the NBS, we re-populated the data sets *N*−1 times by random re-assignment (permutation) of all subjects into one of two groups, where *N* is the number of permutations. We computed *F*-values for the original data set and *N*−1 permuted sets through a simple analysis of covariance, which formed a null distribution of group difference. Then, we estimated the significance level of group difference by a fraction of the occurrence whose *F*-values were not less than the that of the original data set. We used 10 000 as *N*. We also performed the pairwise comparisons using the permutation-based analysis of covariance and the FDR procedure.^51^

## Results

Table 1 shows the demographic and clinical characteristics for the subjects. The 49 MDD patients included five males and 44 females with a median age of 55 years (49–62 years). The median initial HAM-D score was 19, which indicated moderately severe depression. Eight of the forty-nine (16.3%) patients had previously attempted suicide. Patients with suicidal ideation were likely to have attempted suicide and had higher SSI scores. No significant differences in gender, age, education, number of episode, duration of current episode, BIS scores, HAM-D score and MDQ score were present between two patient groups.

The nine healthy males and twenty-two healthy females had a median age of 56 years (range, 51–62 years). The mean level of education of health subjects was 11.40±4.65 years. There was no significant difference in demographic characteristics between the two depressive groups and the healthy subject group.

### Whole-brain mapping of connectivity according to suicidal ideation

**Figure 1:**
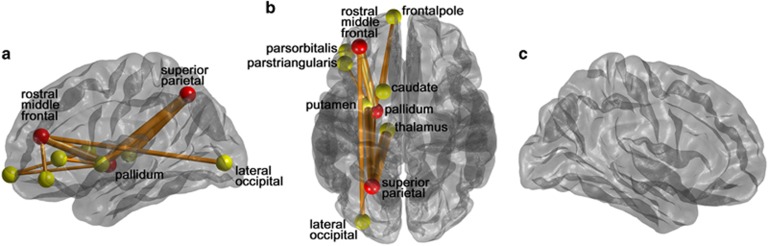
A subnetwork of suicidal ideation in major depressive disorder (MDD) patients identified by the network-based statistical (NBS) analysis shown in the lateral view of the left hemisphere (**a**), the transverse view of the both hemispheres (**b**) and the lateral view of the right hemisphere (**c**). We first compared networks of MDD patients with suicidal ideation and without the ideation using two-sample *t*-test, and identified a subnetwork using NBS (initial threshold was 2.2). The subnetwork consists of significantly reduced connectivity in patients with suicidal ideation. The red circles represent its hub regions representing the brain regions most affected by the white matter disruption, whereas the other yellow circles are the non-hub brain regions. The thickness of edge represents how significantly two groups are different (*t*-statistics).

We characterized a subnetwork whose edge weights decreased in subjects with suicidal ideation compared with the subjects without the ideation in the NBS analysis (Figure 1). The subnetwork included nine edges in the left hemisphere (Table 2) and connected with various subcortical regions (putamen, pallidum and caudate), frontal regions (rostral middle frontal, pars triangularis, pars orbitalis and frontal pole), lateral occipital region and the superior parietal region. There was no significant subnetwork whose edge weights increased in subjects with suicidal ideation compared with the subjects without the ideation. We also determined hub regions of the identified structural network deterioration: the left rostral middle frontal, the left superior parietal and the left pallidum (Figure 1, denoted by red circles).

### Correlations between clinical measurements and connectivity measures

We performed the correlation analysis between the clinical measurement (SSI and three subscales of BIS) and the network topological measures of the hub regions in patients with suicidal ideation (Table 3) using the Spearman partial correlation, controlling effects of age, gender and level of education. The FDR procedure performed over the three hub regions revealed that the betweenness centrality of the left rostral middle frontal was strongly correlated with SSI (*r*=0.59, FDR-adjusted *P*=0.02). The participation coefficient of the same region was significantly correlated with score of attention-cognitive impulsivity of BIS (*r*=0.59, FDR-adjusted *P*=0.01).

We also investigated the correlation between the clinical measurement and edge weight with FDR over nine identified edges reduced in patients with suicide ideation. The score of motor impulsivity of BIS was strongly correlated with edge weight between the left rostral middle frontal and the left pars orbitalis (*r*=0.73, FDR-adjusted *P*=0.001, Table 2), and between the left putamen and the left pars triangularis (*r*=−0.56, FDR-adjusted *P*=0.04, Table 2) in all MDD patients. The whole results of graph analysis were presented in Supplementary Tables 1 and 2.

### Comparison of connectivity between MDD patients and healthy subjects

**Figure 2:**
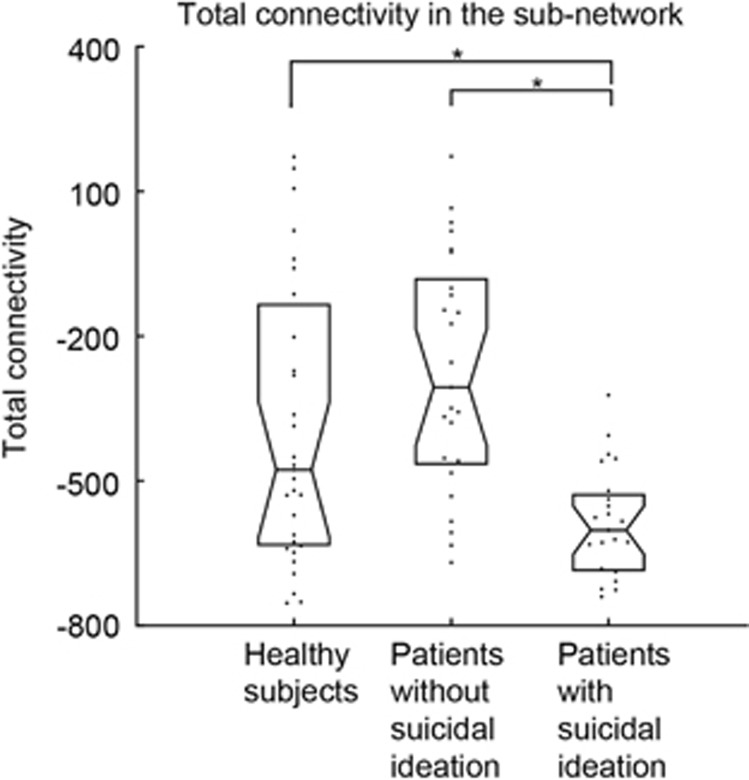
Comparison of total connectivity in the subnetwork of suicidal ideation between major depressive disorder (MDD) patients and healthy subjects. The total connectivity in subjects with suicidal ideation was significantly smaller than the one in subjects without suicidal ideation and normal controls, whereas the one in subjects without suicidal ideation did not differ with the one in the normal controls. We compared the cumulative streamlines in the subnetwork identified by the network-based statistical (NBS) analysis between the healthy subjects, MDD patients without suicidal ideation and with the ideation, controlling age, gender and the level of education (years) using the permutation-based analysis of covariance (ANCOVA). The asterisks represent the significant difference in pairwise comparison (false discovery rate (FDR) adjusted). The values shown here are corrected for the effects of age, gender and the level of education.

The total connectivity (cumulative number of streamlines) of the nine edges in the subnetwork detected in the NBS analysis was significantly different according to the three groups (*P*=0.01, Figure 2). In the pairwise comparison, the total connectivity of nine edges significantly differed between MDD patients with and without suicidal ideation (FDR-adjusted *P*<0.001), and also between the MDD patients with suicide ideation and healthy subjects (FDR-adjusted *P*<0.001). However, there was no significant difference between MDD patients without suicide ideation and healthy subjects (FDR-adjusted *P*=0.47). The results of Kruskal–Wallis rank-sum tests and *post hoc* tests with Bonferroni correction showed the same trends (Kruskal–Wallis tests: *P*<0.0001; MDD with suicidal ideation versus MDD without ideation: *P<*0.0001, MDD with suicidal ideation versus healthy: *P<*0.001 and MDD without ideation versus healthy: *P*=0.29).

## Discussion

To the best of our knowledge, this is the first study comparing connectome-level differences in structural networks between MDD patients with and without suicidal ideation. First, we demonstrated that a distinct brain network characterizes the structural connectivity differences that are present in patients with suicide ideation. This network involves the regions of the left hemisphere, specifically the rostral middle frontal cortex, superior parietal cortex and pallidum. In addition, the network connections consisting of the frontal pole, subdivisions of inferior frontal (pas orbitalis and pars triangularis), lateral occipital, caudate and thalamus were significantly different between the groups. Moreover, we found significant correlations between suicide severity (SSI), impulsivity (attention-cognitive impulsivity and motor subscales of BIS) and network connectivity measures in the subnetwork obtained from the NBS analysis. In adddition, we found this structural network was not significantly different between MDD patients without suicidal ideation and healthy subjects.

We found that the regions involved in executive function were implicated in suicidal ideation in MDD. One of the primary regions apparent from the analysis was the rostral middle frontal, which was one of hub regions in the NBS analysis. Three decreased edges included this region (Table 2). Supplementary Figure S1 is the illustration of different mean edge strengths between left rostral middle frontal and left pallidum according to groups. A significant decrease in this edge strength was observed in the MDD patients with suicidal ideation. The rostral middle frontal region approximates Brodmann's area 10 and is also termed the anterior prefrontal cortex or frontopolar cortex. It is especially developed in humans compared with other primates, and has a critical part in the higher cognitive functions, especially in the integration of executive function.^52, 53^ Another region that showed reduced connectivity was the left superior parietal that is a candidate area outside of the prefrontal cortex, which contributes to executive function.^54, 55^ This finding is in line with previous studies that demonstrated that parietal abnormalities are related to suicidal behavior in psychotic disorder^56^ and MDD.^57^ Impairment of executive function is linked with suicidal ideation or behavior.^58, 59^ Executive function is involved in cognitive flexibility, foresight and weighing possible consequences of behavior, initiation of appropriate actions and inhibition of inappropriate actions.^60, 61^ Impairment of these cognitive actions has clinical relevance for suicide. Our results bolster the view that the differences in structural networks of the region involved in executive function are associated with suicidal ideation. However, our results should be carefully interpreted, as we indirectly investigated association between executive function, suicidal ideation and subnetwork changes due to lack of measurement of executive functions.

We observed reduced connectivity in frontosubcortical circuits involving the edges between the left rostral middle frontal and the left pallidum, between the left frontal pole and the left caudate, and between the left frontal pole and the left pallidum (Figure 1 and Table 2). These circuits involve emotional dysregulation, executive dysfunction, apathy and impulsivity.^62^ Decreased prefrontal activity and activated subcortical function are related with aggressive behavior.^63^ Abnormalities in these circuits may also result in the loss of prefrontal control over the subcortical area. Such disturbances might contribute to the risk for impulsive and aggressive thought.

We found left lateralization related to suicidal ideation. Besides the frontosubcortical circuit, we observed left lateralized reduction of white matter connectivity in the lateral occipital or the inferior frontal (pars orbitalis and pas triangularis). Previous hemispheric asymmetric models^64, 65^ suggest that the left hemisphere is associated with positive emotion and motivation to approach, whereas the right hemisphere is associated with negative emotion and motivation to withdraw. This hemispheric asymmetry has also been studied in relation with impulsivity.^66^ In this respect, impairment in left hemispheric networks could cause suicidal ideation by disturbance in emotion, motivation and impulsivity in depressive patients. However, careful interpretation is needed because our study is not designed to assess asymmetry.^67^

The correlation study, with network measures and hubs, provides a complementary view of the rostral middle frontal region. Its betweenness centrality has a significant positive correlation with the severity of suicidal ideation (Table 3), and the participation coefficient also has a significant positive correlation with motor impulsivity. The betweenness centrality of a given node is defined as the number of shortest paths between any two nodes that pass through this node, and represents the level of influence on the information transformation in the network.^68^ The participation coefficient represents the level of intermodular connectivity,^69^ a brain region with high value of participation coefficient has an important role in communicating the information between modules.^70^ These findings suggest strengthened roles of coordinating brain networks in the rostral middle frontal region in patients with higher suicidal severity or impulsivity, and could be the result of compensation of pathological changes in this region.^31^

We did not find any significant network measure associated with other impulsivity scales; attention-cognitive or non-planning. The attention scale is thought to reflect a person's tendency to shift attention and impatience for complexity; the motor scale is related to impetuous action; and the non-planning scale indicates a lack of consideration of future consequences.^71^ Our results suggested that the brain connectivity that associated with impetuous action is important for suicidality in MDD. This finding is in line with previous reports that bipolar patients with suicidal ideation had higher BIS motor scales than those without suicidal ideation; however, attention and non-planning scale were not significantly different.^72^ However, possibility of false-negative due to the multiple tests and limited sample size should be considered.

Interestingly, the sum of edge strength (total connectivity) involved in suicidal ideation was not different between MDD patients without suicidal ideation and healthy subjects (Figure 2). Thus, the identified subnetwork may be associated with the suicidal ideation, as the groups without suicidal ideation shows no difference in the total connectivity of the identified subnetwork, whereas the group with suicidal ideation significantly differs from the others without suicidal ideation in the total connectivity. In addition, there was no significant difference in depression severity, the number of episodes^73^ nor in the duration of current episodes between two depressive patient groups. A recent DTI study^33^ reported that the default mode network and right frontothalamocaudate regions are prominent in MDD patients; these networks were not significant in the present study. Together with our results, it suggests that the subnetworks are specifically involved in the development of suicidal ideation, and are not associated with depression status or severity of the disorder. One possible speculation is that patients with premorbid reduced connectivity in the subnetwork that we found are more vulnerable to develop suicidal ideation when they experience depressive episodes.

On the other hand, this result raises a question that the suicidal ideation has its own neurological changes independent of specific psychiatric disorders. Previous studies of other disorders denoted differences in its own specific brain regions in patients with suicidal ideation. A DTI study reported that the fractional anisotropy of the right cingulum was positively correlated with current suicidal ideation in traumatic brain injury.^74^ In functional MRI studies, suicidal ideation was negatively correlated with left striatal activation in bipolar II depression patients.^75^ Moreover, combat-exposed war veterans with suicidal ideation showed more error-related activation of the anterior cingulate and prefrontal cortex.^76^ Although the methodological difference between our study and previous reports exist, the anatomical regions found in these studies were not consistent. It suggests that multiple regions would be related to suicidal ideation, and the pathophysiology of psychiatric disorders would interact with this relationship.

Recent reports of a rapid antidepressant action of the glutamate *N*-methyl-*D*-aspartate receptor antagonist ketamine highlight a novel intervention.^77^ One of the major advantages of ketamine as an antidepressant is its' remarkable suppression of suicidal ideation.^78^ The mechanism of this action is unclear; however, activation of the mammalian target of rapamycin leading to increased spine density and synaptic activity in the prefrontal cortex was suggested as a candidate pathway of the rapid onset of the effect of ketamine.^79^ Further studies of the effect of ketamine on the prefrontal region of the subnetwork found in our study might be helpful to reveal the detailed mechanism.

Several limitations should be noted. First, the study was cross-sectional; therefore, it was difficult to verify the causal relationship between suicidal ideation, impulsivity and white matter changes. Second, possibility of gender and age bias should be noted. Our patients were mostly female and matured age, therefore the generalizability of our results to depressed patients in other gender- and age-group may be limited. Third, we could not verify the effect of white matter change on the outcome of suicidal death. Fourth, we could not include some variables that might be underlying causes of the altered connectivity, such as history of early trauma^80^ and history of previous suicide attempts,^21 22^ due to lack of information and the limited sample size. Further studies with a larger population with longitudinal design and controlling diverse covariates are warranted.

Lastly, our diffusion MRI data are rather suboptimal with respect to fiber tracking. In particular, the voxel dimensions are anisotropic (2 versus 1.72 mm), which can bias tracking accuracy in the longer *z* direction. As it is only 16% longer than the other directions; however, this bias might be insignificant. Our study inherited the limitation of DTI and the deterministic tractography, including the issue of crossing-fibers. Other tracking methods such as probabilistic tractography^81^ or Hough Transform global tractography^82^ could be employed in future research.

In summary, we describe a subnetwork that characterizes suicidal ideation in MDD patients. This subnetwork includes frontosubcortical circuit and regions involved in executive function. The network measures in this subnetwork were correlated with suicide severity or impulsivity. In addition, the differences of this subnetwork according to suicidal ideation are independent of depressive status. These results provide the first evidence of white matter connectivity changes in suicidal ideation in MDD patients.

## Figures and Tables

**Table 1:** Clinical and demographic characteristics of subjects

*Characteristics*	*Total*	*Suicidal ideation (*n*=24)*	*No suicidal ideation (*n*=25)*	*Healthy controls (*n*=31)*	P
Gender, male (%)^a^	14 (17.5%)	3 (12.5%)	2 (8.0%)	9 (29.0%)	0.13
Age, years^b^	55.5 (50, 62)	55.5 (46, 62)	55 (50, 60)	56 (51, 62)	0.85
Education, years^c^	11.29±4.12	11.42±4.62	11.16±3.66	11.40±4.65	0.91
Previous attempt history (%)^a^	8 (16.3%)	8 (33.3%)	0 (0%)	—	0.002
Number of episodes^d^	2 (1,2)	2 (1,2)	1 (1,2)	—	0.15
Duration of current episode, years^d^	0.6 (0.2, 1.7)	0.6 (0.2, 1.85)	0.6 (0.2, 1.6)	—	0.94
SSI^d^	10 (2, 16)	15.5 (10, 23.5)	3 (0, 12)	—	<0.0001
					
*BIS*					
Motor^e^	15.69±3.97	15.88±4.08	15.52±3.94	—	0.97
Attention-cognitive^e^	15.96±3.09	15.54±3.05	16.36±3.13	—	0.79
Non-planning^e^	20.27±5.09	20.00±5.16	20.52±5.12	—	0.98
HAM-D^d^	19 (17, 22)	20 (17.5, 23)	18 (17, 20)	—	0.12
MDQ^d^	4 (1, 7)	3.5 (1, 7.5)	4 (2, 6)	—	0.89

Abbreviations: ANOVA, analysis of variance; BIS, Barrett Impulsiveness Scale; HAM-D, Hamilton Depression Rating Score; MDQ, Mood Disorder Questionnaire; SSI, Scale for Suicide Ideation.

a Fisher's exact test was used.

b Kruskal–Wallis test was used; data are given as the median and interquartile range.

c One-way ANOVA was used; data are given as the mean and s.d.

d Wilcoxon rank-sum test was used; data are given as the median and interquartile range.

e Student's *t-*statistics was used; data are given as the mean and s.d.

**Table 2:** Reduced connectivity in depressive patients with suicidal ideation found using the NBS analysis compared with depressive patients without ideation

*Edges*	*t-statistics*^a^	*Mean edge strength*		*Correlation coefficients in MDD with suicidal ideation*^b^			
		*Suicidal ideation*	*No suicidal ideation*	*SSI*	*BIS motor*	*BIS attention-cognitive*	*BIS non-planning*
Left rostral middle frontal^c^ and left pallidum^c^	3.01	5.04	40.68	−0.24	−0.08	−0.28	−0.27
Left rostral middle frontal^c^ and left lateral occipital	2.52	0.54	5.32	0.23	−0.13	0.27	0.27
Left rostral middle frontal^c^ and left pars orbitalis	2.44	50.67	98.40	0.40	**0.73**^d^	0.20	0.21
Left superior parietal^c^ and left thalamus	3.21	14.08	58.52	0.25	0.21	0.02	0.17
Left superior parietal^c^ and left putamen	2.94	81.25	162.76	−0.15	−0.04	−0.22	−0.10
Left superior parietal^c^ and left pallidum^c^	2.25	17.00	41.60	−0.44	−0.39	−0.26	−0.42
Left pars triangularis and left putamen	2.37	27.75	78.16	−0.27	**−0.56**^d^	0.13	−0.19
Left frontal pole and left caudate	2.37	10.50	43.12	−0.18	0.00	−0.29	−0.21
Left frontal pole and left pallidum^c^	2.34	0	3.92	NA	NA	NA	NA

Abbreviations: BIS, Barrett Impulsiveness Scale; FDR, false discovery rate; MDD, major depressive disorder; NA, not available; NBS, network-based statistics; SSI, Scale for Suicide Ideation.

a Student's *t*-statistic from two-sample *t*-test, (MDD_without_−MDD_with_).

b Correlation coefficients between edge weights and SSI/BIS scores in MDD with suicidal ideation.

c Hub nodes.

d Bold, significant after FDR correction over nine edges.

**Table 3:** Significant correlations between clinical variables and connectivity measures in patients with suicidal ideation (FDR-adjusted *P*<0.05)^a^

	*Clinical measurements*	r	*FDR-adjusted* P
*Betweenness centrality*			
Left rostral middle frontal	SSI	0.59	0.02
			
*Participation coefficient*			
Left rostral middle frontal	BIS (attention-cognitive)	0.59	0.01
			
*Edge weight*			
Left rostral middle frontal and the left pars orbitalis	BIS (movement)	0.73	0.001
Left putamen and the left pars triangularis	BIS (movement)	−0.56	0.04

Abbreviations: BIS, Barrett impulsiveness scale; FDR, false discovery rate; SSI, Scale for Suicide Ideation.

^a^Spearman partial correlation coefficients controlling age, gender and level of education.
