# Supplementary material for: Reduced frontal-subcortical white matter connectivity in association with suicidal ideation in major depressive disorder
Source: Transl Psychiatry. 2016 Jun 7;6(6):e835–. doi: 10.1038/tp.2016.110 (PMC4931608; doi:10.1038/tp.2016.110)
Supplement: Supplementary Tables 1 and 2 [file tp2016110x1.pdf]

Supplementary Table 1. Correlation analysis between the clinical measurement (SSI and three subscales of BIS) and the network topological measures in patients with suicidal ideation

	Nodal degree			Nodal strength			Clustering coefficient			Participation coefficient			Regional efficiency			Betweenness centrality		
	r†	p	adj. p‡	r†	p	adj. p‡	r†	p	adj. p‡	r†	p	adj. p‡	r†	p	adj. p‡	r†	p	adj. p‡
Scale for Suicide Ideation																		
Left rostral middle frontal	-0.164	0.478	0.935	0.306	0.177	0.530	0.427	0.053	0.160	0.065	0.778	0.778	0.196	0.394	0.563	0.587	0.005	**0.016**
Left superior parietal	0.055	0.813	0.935	0.108	0.642	0.964	0.174	0.451	0.657	-0.100	0.667	0.778	0.134	0.563	0.563	0.193	0.402	0.603
Left pallidum	0.019	0.935	0.935	0.003	0.989	0.989	-0.103	0.657	0.657	-0.185	0.421	0.778	0.203	0.378	0.563	N/A	N/A	N/A
Motor impulsivity																		
Left rostral middle frontal	-0.163	0.481	0.585	0.054	0.815	0.815	0.151	0.514	0.514	-0.138	0.551	0.826	0.184	0.425	0.454	-0.159	0.492	0.738
Left superior parietal	-0.197	0.391	0.585	0.123	0.594	0.815	0.307	0.176	0.264	-0.362	0.107	0.322	0.173	0.454	0.454	-0.163	0.482	0.738
Left pallidum	-0.126	0.585	0.585	0.224	0.329	0.815	0.497	0.022	0.066	0.047	0.838	0.838	0.445	0.043	0.129	N/A	N/A	N/A
Attention-cognitive impulsivity																		
Left rostral middle frontal	-0.184	0.426	0.689	0.018	0.940	0.940	0.151	0.513	0.921	0.589	0.005	**0.015**	-0.010	0.967	0.967	0.246	0.283	0.609
Left superior parietal	-0.093	0.689	0.689	-0.094	0.685	0.940	-0.048	0.836	0.921	-0.137	0.553	0.615	0.032	0.891	0.967	-0.191	0.406	0.609
Left pallidum	-0.140	0.546	0.689	-0.064	0.784	0.940	-0.023	0.921	0.921	0.116	0.615	0.615	0.090	0.698	0.967	N/A	N/A	N/A
Non-planning impulsivity																		
Left rostral middle frontal	-0.294	0.196	0.342	-0.124	0.593	0.971	0.087	0.707	0.818	0.438	0.047	0.141	-0.066	0.777	0.902	0.004	0.985	1.478
Left superior parietal	-0.205	0.373	0.373	-0.070	0.764	0.971	0.053	0.818	0.818	-0.064	0.781	0.781	0.029	0.902	0.902	-0.204	0.375	1.124
Left pallidum	-0.275	0.228	0.342	-0.008	0.971	0.971	0.214	0.351	0.818	0.123	0.596	0.781	0.172	0.456	0.902	N/A	N/A	N/A

Abbreviation: BIS, Barrett Impulsiveness Scale

†Spearman partial correlation coefficients after controlling effects of age, gender, and level of education.

‡FDR (False Discovery Rate) adjusted p-value where the FDR procedure was performed over the three hub regions. Bold represents a significant result after FDR adjustment.

Supplementary Table 2. Correlation analysis between three subscales of BIS and edge weights in all patients

	Motor impulsivity			Attention-cognitive impulsivity			Non-planning impulsivity		
Edge	r†	p	adj. p‡	r†	p	adj. p‡	r†	p	adj. p‡
Left rostral middle frontal and left lateral occipital	0.330	0.025	0.106	0.355	0.015	0.070	0.237	0.112	0.358
Left rostral middle frontal and left pars orbitalis	0.347	0.018	0.106	0.357	0.015	0.070	0.233	0.119	0.358
Left superior parietal and left thalamus	0.119	0.429	0.773	0.020	0.893	0.952	-0.017	0.913	0.996
Left frontal pole and left caudate	-0.021	0.892	0.892	-0.096	0.526	0.789	-0.174	0.247	0.556
Left pars triangularis and left putamen	-0.049	0.748	0.842	0.044	0.772	0.952	-0.060	0.690	0.887
Left superior parietal and left putamen	-0.311	0.035	0.106	-0.160	0.288	0.789	-0.362	0.013	0.120
Left rostral middle frontal and left pallidum	-0.079	0.602	0.842	-0.101	0.503	0.789	-0.082	0.586	0.879
Left superior parietal and left pallidum	-0.123	0.414	0.773	0.009	0.952	0.952	-0.099	0.514	0.879
Left frontal pole and left pallidum	0.058	0.701	0.842	-0.128	0.396	0.789	0.001	0.996	0.996

Abbreviation: BIS, Barrett Impulsiveness Scale

†Spearman partial correlation coefficients after controlling effects of age, gender, and level of education.

‡FDR (False Discovery Rate) adjusted p-value where the FDR procedure was performed over the three hub regions.
