# Supplementary figures and images for: Reduced frontal-subcortical white matter connectivity in association with suicidal ideation in major depressive disorder
Source: Transl Psychiatry. 2016 Jun 7;6(6):e835–. doi: 10.1038/tp.2016.110 (PMC4931608; doi:10.1038/tp.2016.110)

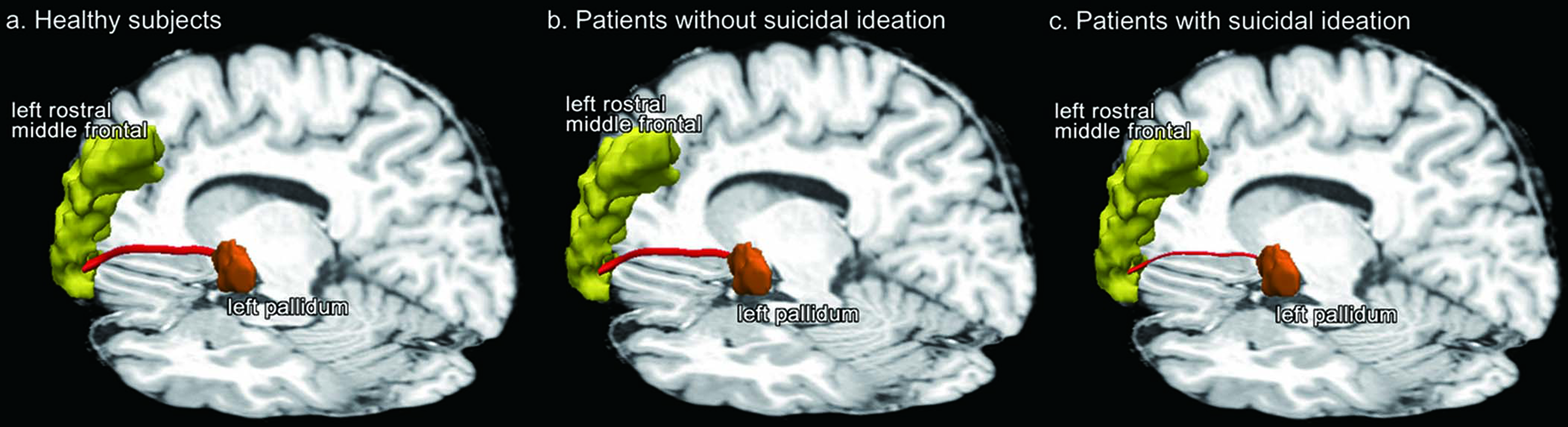

Supplement: Supplementary Figure S1 [file tp2016110x2.tif]
